# Four-step approach to idea management sequencing: redefining or reinventing values in a business model

**DOI:** 10.1186/s13731-022-00236-1

**Published:** 2022-09-29

**Authors:** Elina Mikelsone, Inga Uvarova, Jean-Pierre Segers

**Affiliations:** 1 Idea Innovation Institute, Tomini, Cenu Parish, District of Jelgava, 3018 Latvia; 2 BA School of Business and Finance, Kr. Valdemara Street 161, Riga, 1013 Latvia; 3 ArtSmart Ltd., Stepini, Aizkraukles nov., 5101 Latvia; 4 Riga Technical University, Kalnciema Street 6, Riga, 1048 Latvia; 5 Hasselt University, Agoralaan (Building D), 3590 Diepenbeek, Belgium

**Keywords:** Idea management, Sequencing, Business model, Value proposition, Ideation, Design thinking

## Abstract

The purpose of this paper is to create and test an idea management sequence framework to reinvent or redefine the value proposition. Idea management with sequencing activities must be considered as a systematic managerial process and should not be confused with the occasional result of an individual with a design thinking mindset. This paper suggests a new approach—a systematic, 4-step idea management sequence to redefine or reinvent value proposition in a business model, which was validated through an action-based research method involving 20 managers from practice by applying the proposed framework. Based on the idea management approach, authors describe the idea generation and evaluation processes and their possible moderation elements. This research contributes to previous studies of the design thinking and innovation by substantiating a concept of the idea management sequencing and proposing a new 4-step approach that can be applied by organisations to redefine or reinvent value proposition in their business models. Being influenced by pandemic restrictions and the full or partial remote workforce, the 4-step idea management approach is beneficial for virtual group sessions as it increases the quality of outcomes, engagement of individuals, collaborative openness, and confidence.

## Introduction

Since the start of the twenty-first century design thinking has emerged in various fields of the social sciences (Baker & Moukhliss, 2020), including such practices as discovery, ideation, prototyping, testing, and experimentation (Jaskyte & Liedtka, 2022). Being closely interrelated with the design thinking, the field of idea management (IM) has grown in relevance over the last decade. Both concepts are linked with various innovation approaches, for instance, system thinking, understanding the perception of the value and value mapping, mission innovations and “moonshots”—disruptive innovations (Allen, 2022; Geissdoerfer et al., 2016; Mazzucato, 2021). The IM and its web-based solutions are not just about the creation of new ideas or capabilities to ideate or innovate, but they embrace the managerial process of systematic activities for exploring or recognising needs, generating or creating variations, evaluating and measuring the survival of new ideas, also, taking decision on their adoption and realisation (Vandenbosch et al., 2006).

The level of idea generation and innovation can vary significantly within a single country or even a region, so these differences are not only influenced by economic factors or the entrepreneurial activity but are largely determined by the behavioural patterns of the people involved in the innovation and its processes (Soloviov, 2022), the notion of the design thinking mindset (Venkatesh et al., 2012) and facilitation of the IM as a systematic and sequenced managerial process (Allen, 2022; Meslec et al., 2020). Previous studies confirm that the ideation is an essential part of the design thinking (Micheli et al., 2018), but the conceptual settings part of managerial strategies and approaches of the ideation and IM sequence within organisations and teams are less conceptualised by the academic community (Meslec et al., 2020). Ideation has a dynamic, agile, and non-static nature that poses challenges to maintain the systematic and sequencing nature in IM.

The evolution of design thinking and the IM movement occurred in parallel with the development of theoretical concepts of the value chain (Porter, 1985) and the business model (Magretta, 2002; Zott et al., 2011). The latter two, to a large extent, exploit the design thinking and the IM methods for generating, redesigning, testing, and experimenting with new business models and values.

The interpretation of the value concept has expanded significantly in the context of the various characteristics of a business model (DaSilva & Trkman, 2014). An important theoretical basis for the value has been outlined by Porter (1985) with the competition theory, where the value is related to the business processes and activities that support organisations in achieving a competitive advantage.

Since the early 2000s, design thinking has grown in academic topicality, leading to an increased design thinking application to novel challenges (Baker & Moukhliss, 2020). In this study, the developed 4-step IM sequencing approach will be applied to define the value proposition for a company. One of the main causes of new ventures’ failing is associated with the lack of viable ideas or an inability to develop business models to realise new ideas and bring them to market (Allen, 2022). This indicates problematic challenges related to overall perception of the importance of the ideation and the design thinking, and the consequent need for IM within organisations.

In this paper, the authors apply the definition that the design thinking is a human-centred approach that includes the generation of many ideas, and the adoption of a fast-prototyping approach (Foster, 2021). In this research, various design thinking approaches are exploited, for example, a persona method identifying needs and desires of customers, users and other stakeholders (Chasanidou et al., 2015; Stickdorn & Schneider, 2010) and also creative thinking methods, like, Mind Mapping (Wycoff, 1991), trend watching (Trendwatching, 2021) and other methods.

The idea is considered as the starting position for inventing something new, reinventing or maintaining the status quo (Vandenbosch et al., 2006). The IM helps to provide more effective and efficient idea generation, evaluation, and selection processes (Brem & Voigt, 2007). There are various brainstorming methods to support the idea generation (Bonnardel & Didier, 2020). The authors have described the idea generation and evaluation processes based on the IM approach and have applied design thinking to create the sequence of the approaches incorporated into the IM process.

The IM approach does not limit itself to design thinking, it is used in the system thinking and other different innovation approaches. Organisations have sought for systematic IM approaches to help them in defining and redefining their value proposition. This paper provides a new sequence of the IM as the answer to this demand. Design thinking seems to play an important role in innovating and establishing a successful new business model (Guldmann et al., 2019; Sokolic, 2015), therefore authors would like to research more in-depth the IM as an important element within this context. Another rationale is that it is a human-centred approach that includes the generation of many ideas, and the adoption of a fast-prototyping approach (Foster, 2021).

In the researched case, an organisation aims to create a larger number of versions of new ideas for the value proposition, then describe and verify value definitions by involving various stakeholders. Based on the IM approach, authors describe the idea generation and evaluation processes by applying the design thinking approach and their possible moderation elements that could be appertained to an organisation to find a new or reinvent an existing value proposition.

**Fig. 1:**
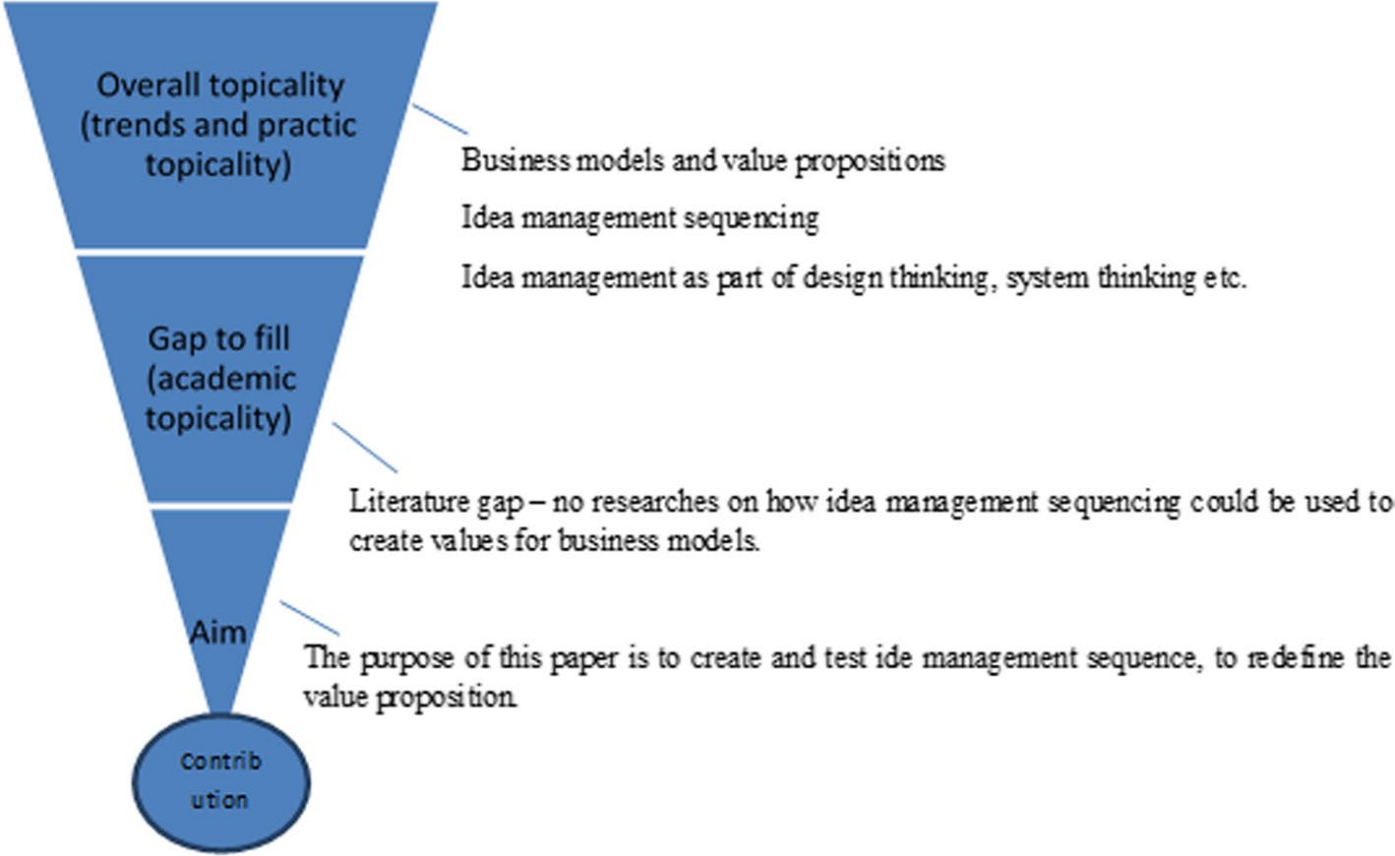
Research categories, gaps and motivation (source: the authors)

Authors fill the research gaps described above and as visualised in Fig. 1 by applying a theoretical literature review and the empirical approach with the case study and the action research (Blessing & Chakrabarti, 2009), whereas the last is the methodological novelty in this discipline (Shapira et al., 2017).

The purpose of the paper is to create and test the IM sequence to redefine or reinvent the value proposition by presenting a 4-step approach of the systematic IM sequence.

There are several contributions: (1) the IM construct characterised in the context of the value proposition of the business model; (2) the IM sequencing as the managerial process is substantiated and linked within the context of the business models and values; (3) the practical sequence of the IM is created and tested with the proposed 4-step approach.

The 4-step approach was adopted to current Covid-19 pandemic circumstances with full or partial remote work conditions in mind. The 4-step approach sessions have been adapted to the virtual environment using various digital tools to moderate and encourage group contribution to the ideation process. The proposed method uses the IM approach, in which it is extremely important for all stakeholders to engage in an active ideation process and keep their attention, engagement and trust of remote participants in the virtual sessions. The approach is linked to the digital context and provides the ability to run experimental and brainstorming sessions across multiple iterations in a digital environment (Magistretti et al., 2020).

## Theoretical background

### Idea management and sequencing

In this paper, the definition of the IM is a systematic and manageable process with two main parts: (1) the idea generation and the evaluation, and (2) repeated idea generation and evaluation if it is needed (Miķelsone et al., 2019). Thus, the essential characteristic factors that distinguish IM process from the eventual, uncontrolled, or philosophical idea creation process is that the IM is considered as a managed process, function, or an activity. IM is a systematic approach with several stages or processes of idea generation and creation that is followed by idea evaluation, measuring and continuation of this process (Vandenbosch et al., 2006).

Table 1 demonstrates the main elements describing stages of the IM. From the perspective of the managerial process, the IM represents three stages (Table 1). These stages can be considered as the toolkit or a complex system of the managerial process in order to facilitate the IM.

**Table 1:** Idea management stages (IMS). Source: developed by the authors based on Mikelsone et al. (2021)

1st stage—generation	2nd stage—evaluation	3rd stage continuation
Idea generation—exploring the need, preparation, capture/gathering of ideas, retention, enhancement	Idea evaluation—screening, selection, retention	Continuation of IM—concept development, distribution of ideas, support during implementation with repeated IM and rewarding, retention
Korde and Paulus (2016), Wooten and Ulrich (2015), Summa (2004)	Westerski (2013), Summa (2004)	Summa (2004)

*Additional characteristics* These systems provide parallelism: the ability for members to exchange information simultaneously (Dennis & Garfield, 2003), anonymity (which enables members to make contributions without attaching their names which is not possible when contributions are made verbally (Dennis & Garfield, 2003), transparency (transparent evaluation process (Summa, 2004) and are applicable for different kind of idea generation and evaluation aims

In this paper, authors concentrate on the first two stages (Table 1), also describing possible versions of the 3rd stage for the continuation of the IM.

Scholars distinguish 3 IM types depending on the human resources involved in the IM process: external, internal and mixed IM. The external IM means that external idea generation and evaluation takes place (main IM sources: experts, partners, customers and other stakeholders (outside of the organisation) (e.g. Bothos et al., 2008, 2012; Tung et al., 2009; Westerski & Iglesias, 2011).

The internal IM is internal idea generation and evaluation in an organisation exploiting internal resources (employees) of the organisation (e.g. Aagaard, 2012; Bansemir & Neyer, 2009; Bassiti & Ajhoun, 2013; Bettoni et al., 2010; Deichmann, 2012; Fatur, 2007; Glassmann, 2009; Iversen et al., 2009; Klein & Lechner, 2010; Moss et al., 2011; Poveda et al., 2012; Selart & Johansen, 2011; Shani & Divyapriya, 2011; Wood, 2003).

In mixed IM, the idea generation and evaluation involve both internal and external sources (e.g. Baez & Convertino, 2012; Brem & Voigt, 2007, 2009; Enkel et al., 2009; Fritz, 2002; Nilsson et al., 2002; Sandstrom & Bjork, 2010; Voigt & Brem, 2006). See in Table 2, the comparison of the 3 IM type classification.

**Table 2:** Idea management types. Source: developed by the authors based on Mikelsone et al. (2020)

Classifications			
Classification criteria: based on the application focus			
Passive IMS		Active IMS	
*Functions* Focus on idea generation	*Type of focus* Unfocused process	*Functions* Focus on all IM dimensions	*Type of focus* Focused process

Classification criteria: based on the involved IM source					
Internal IMS		External IMS		Mixed IMS	
*Description* IMS that allows involving only internal IM sources	*Main IM source* Employees	*Description* IMS that allows involving only external IM sources	*Main IM source* Crowds, experts, clients, etc	*Description* IMS that allows involving internal and external IM sources	*Main IM source* Employees; clients, experts, crowds, etc

**Fig. 2:**
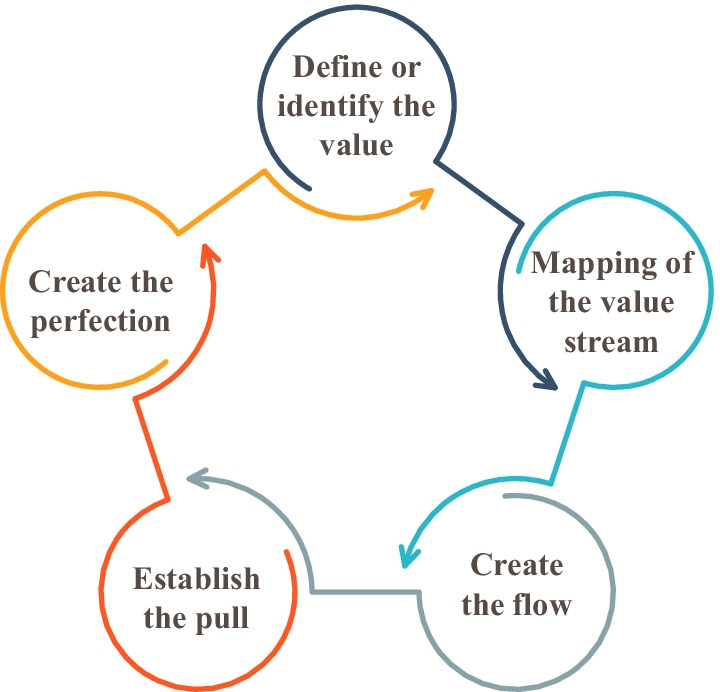
Sequence of Lean principles (source: created by the authors based on Allen, 2,022)

The IM sequencing is a new term which has not been yet actively described by scholars, but in recent years it has been gradually receiving attention from researchers. It is defined as analysing design thinking and ideation from the perspective of a systematic process with interrelated and sequent steps, adding value to the result. Allen (2022) uses the Lean concept to describe the sequence within the design thinking and separates several Lean principles that have a certain nature of sequent stages, all of which together form a process that leads to a result or bring a value. The concept of value in the context of Lean has a slightly different meaning, focusing more on adding value to customer and preventing losses, including unnecessary steps in the process (waste) (Gülyaz et al., 2019). However, in a broader interpretation of value, this process can establish similarities with the value in the context of a business model that envisages the value creation, delivery, proposition, and capturing (Segers et al., 2021). This Lean multi-step procedural approach in its essence aims to create value that has similarities with the creation of a new or improved value proposition with the IM sequencing 4-step approach proposed in this study (Fig. 2).

The concept of sequencing is used also in the project management discipline for scheduling the sequence of tasks or activities to be performed within a project (Maxfield, 1981). The project management perspective applies to this study as it proposes the managerial perspective on how to organise several tasks, steps or activities into the network or path leading to the result, so called a sequence-based approach (Shi et al., 2014), which in the case of this study is the definition of new value proposition using the IM approach. As the IM is a dynamic and not a static process, it may not just entail a linear sequence of stages, but also the review and repetition if the result does not meet expectations.

In this study, the sequencing approach is considered for the IM within teams of individuals. When covering the sequencing approach on the level of individuals, it leads academics to discuss behavioural factors. Jimenez-Liso et al. (2022) discuss the instructional sequencing for student engagement. The instructional sequence was used in group works to generate and share ideas and hypothesis, evaluating their experimental designs. According to Jimenez-Liso et al. (2022), the instructional sequence stimulated individuals’ interest, concentration, satisfaction and joy in the process and about the results and co-working, thus increasing and maintaining a high level of engagement throughout. Researchers proved the relationship where the interest and satisfaction lead to the confidence of individuals involved in the brainstorming and discussion sessions (Jimenez-Liso et al., 2022). It is an essential and a beneficial insight that motivates researchers to consider the use of the sequencing-approach within ideation and the IM process.

In the context of the design thinking, the deliberate sequencing with the diversified content and type of tasks increases the creativity of groups or individuals taking part in the idea generation (Meslec et al., 2020). The most important conclusion from researchers (Meslec et al., 2020) in the context of this study is that the sequencing alone does not provide a higher level of creativity and results of the idea generation. When planning the sequence of ideation tasks, it is important to anticipate different types and contents of tasks from a wide range of design and creative thinking methods.

The IM sequencing assumes previously specified presumptions where the sequencing approach leads to higher engagement and confidence, and the combination of the sequencing approach with a variety of the design thinking methods that facilitates better creativity results from the ideation process. In addition, the IM sequencing provides the systematic management of such processes as—the generation of new ideas and their evaluation.

### Business models and values

The concept of business models is integrated with a variety of academic disciplines (Chesbrough & Rosenbloom, 2002), for example, innovation and strategy (Magretta, 2002; Schwarz & Legner, 2020; Vanhaverbeke & Chesbrough, 2014), business architecture (Gassmann et al., 2014), entrepreneurial analysis (Schaltegger et al., 2016), tooling exploration (Athanasopoulou & De Reuver, 2020) and digitalisation (Witschel et al., 2019).

The three common pillars of business models are: (1) value proposition, (2) value creation/delivery, and (3) value capture (Segers et al., 2021). In this article, authors identify the opportunities: (1) value propositions created with IM sequencing.

The topicality of business model studies and how to create value by involving different stakeholders in business model creation (Andreassen et al., 2018; Gay, 2014; Simberova & Kita, 2020) has grown from both academics and practitioners’ sides. The large stream of research on business models (Wirtz et al., 2016) converges with the idea that a business model offers holistic and systemic insights. The sum of complementary elements in business models is: (1) value proposition, (2) value creation/delivery, and (3) value capture.

The concept of business models is integrated with a variety of academic disciplines (Chesbrough & Rosenbloom, 2002), such as business architecture (Gassmann et al., 2014; Teece, 2010), innovation and strategy (Magretta, 2002), interconnected and interdependent activity systems (Zott et al., 2011), value generation (Osterwalder & Pigneur, 2010; Osterwalder et al., 2005), open innovation (Vanhaverbeke & Chesbrough, 2014); Podmetina et al., 2017), managerial and entrepreneurial analysis unit (Schaltegger et al., 2016). Amit and Zott (2020) stress the fit between the business model, the classic strategy (strategic fit), the organisation (internal fit), and the ecosystem (external fit). Porter and Kramer (2011) added the concept of shared value, resulting from policies and practices that contribute to competitive advantage while strengthening the communities in which a company operates. Segers et al. (2021) propose a typology of ten business model families, including IM business model. IM is, without a doubt, connected to business model development, identifying new business opportunities to fit or change the business model and value proposition.

Since 2010s, the design thinking methods are linked to the scientific discourse on the value creation, delivery and capturing in a sustainable and circular business model (Gay, 2014; Geissdoerfer et al., 2016; Uvarova et al., 2020). Comparing to traditional business models, sustainable business models assume the value proposition not just to customers, but also to various stakeholders, incorporating economic, social, and environmental values (Bocken et al., 2013; Geissdoerfer et al., 2016) thus reflecting the sustainable transition process (Jonker et al., 2020; Uvarova et al., 2021). It shows the necessity to reach a wider range of stakeholders by the IM process within and beyond the formal structures of an organisation.

According to Geissdoerfer and his co-authors (2016), the use of design thinking methods in the value innovation process provides an opportunity to create new types of value, as well as to expand the range of different stakeholders to whom the value proposition can be addressed. At present, the value innovation is not only an issue for some practitioners, but an important priority for the top management of companies, where creative and design thinking methods play a promising role as it offers an effective approach for the ideation of new values (Leavy, 2010).

**Fig. 3:**
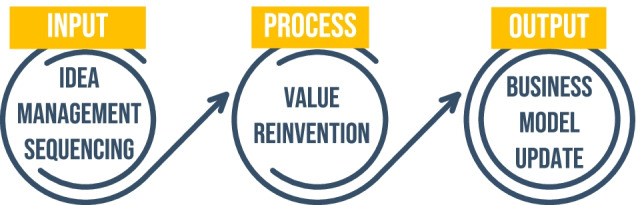
The theoretical framework of this research (source: the authors)

The main contribution is filling the research gap by connecting all three elements as shown in Fig. 3.

### Methodological framework

**Fig. 4:**
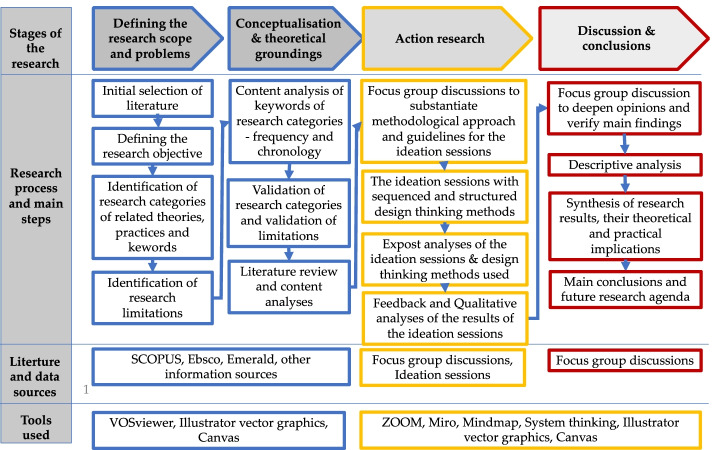
The methodological framework of this research (source: the authors)

This research is based on a qualitative research method, combining the literature review and the action-based research with the ideation sessions using design thinking methods in the focus group discussions, and a descriptive analysis to synthesise the results of the research, implications and future research issues. The methodological framework of this research is presented in Fig. 4, illustrating the main stages of the research, the literature, and data sources, as well as digital tools used.

The action-based research allows the experimentation with the theory in real world organisations and deepen the views and opinions about the enablers and obstacles of the intervention, the solutions or activities performed (Somekh, 2005). This combination of the theory and practice is done with the simultaneous interaction between researchers and practitioners, ensuring the co-work within the sequenced activities of the situation analyses, the experimentation, and the systematic intervention activities. This is achieved by analysing and describing the practices applied, gathering the feedback, and reviewing the lessons learned (Avison et al., 1999). The action-based research provides the methodological framework for researching the dynamic situation and how innovation processes function in these environments (Somekh, 2005). This type of research is particularly relevant to current circumstances as the organisations and their surrounding environment has experienced significant changes brought on by the Covid-19 pandemic and leading to new forms of partial or full remote work, a rapid digital leap forward, but also a step backwards from the increased social distance between employees, customers, and other stakeholders. The European countries encounter the green transformation towards sustainable development goals that aim to change the lifestyle and consumption behaviour of the society, the value orientation of organisations, foster the emergence of a new ecosystem with the open cooperation of various stakeholders addressing the sustainability issues. The action-based research provides the possibility to test the feasibility and nature of new ideas (Kaplan, 1998) that in the context of this study ensured greater options to test and advocate new values generated during the interaction sessions with involved participants. The involved participants later become the knowledge ambassadors or more “skilled implementers” (Kaplan, 1998—1p.) that can promote both new values and new skills of innovating these values within their organisations.

According to Somekh (2005), this study assumed eight methodological principles of the action-based research as presented in Table 3.

**Table 3:** Methodological principles of the action research applied within this study. Source: developed by the authors based on Somekh (2005)

Reasons and methodological principles of the action research
1. The combination of research and action
2. Collaborative partnership of researchers and participants or so called “insiders” of the case
3. The development of knowledge and understanding of a particular case
4. Action research starts from a vision of social transformation and personal engagement of individuals representing the organisation
5. Action research involved a high level of reflexivity and sensitivity of individuals influencing the whole research process
6. Action research involves exploratory engagement with a wide range of existing and interdisciplinary knowledge testing its usefulness
7. Action research evokes learning for participants through combining research, actions, and reflection of the practice
8. Action research requires a deep understanding of the broader historical, political, economic, and ideological contexts shaping the behaviour of individuals

Within this study, we combine the principles of the action research with the IM as they are closely related and foresee the active involvement of participants in the co-creation (Stier & Smit, 2021) and the ideation process (Hesmer et al., 2011; Meslec et al., 2020) of new values. This methodological approach allows better knowledge valorisation (Stier & Smit, 2021) to utilise the academic knowledge within the co-creation of the value innovation by application of IM methods.

The case study was conducted in a medium-sized company located in Latvia with over 50 employees (according to the EU recommendation 2003/361). The company works in the field of innovation and investment consulting. The company stated a necessity to redefine their value propositions. During the 8-h session, 20 managers were involved, but additionally it was required to receive ideas and evaluation from other employees and partners. The case study process was defined in several process steps as seen in Table 4.

**Table 4:** Case study steps. Source: developed by the authors

Data gathering method	Data analysis method	Time period	Method application steps
Action research of the case	Content analysis	2021	1. The preparation for a session to redefine the values 2. A pre-session 3. A practical session moderation 4. A post-session 5. The desk review of documents and information gathered within a practical session 6. The content analysis of materials of a practical session 7. The descriptive analyses of the preparation, performance and the evaluation of a practical session

The preparation for a session aimed to redefine the value proposition, and it included 2 meetings within the organisation. The first meeting was organised to understand the company’s needs in detail, the second one to approve a session plan. Before the second session, detailed research on possible approaches of design thinking was carried out to reach the aims of the company. The authors have evaluated over 20 approaches to select and combine the approach to reach the aim.

In a pre-session, prior to the first meeting, an additional issue was discovered that during an 8-h session, only 20 managers of the company could take part, but the company demanded the additional involvement of more than 100 employees and partners. That was the reason the research team created the pre- and post-sessions. During the pre-session, the list of over 50 values that were mentioned in the company’s documents, strategies and normative acts was created and given for evaluation to the employees. So, the session started with the development of highly evaluated values. The preparation of the value list itself was separate research that is not described in this paper. The post-session was conducted to evaluate and improve the created definitions of the value propositions. This is an additional recommendation for a moderation—if, during a main session resources do not allow to involve all possible stakeholders, there is a possibility to create a pre (generation) and post (evaluation) sessions. Lastly, the authors describe the sequence of the created and tested practical session.

## Results

The 4-step approach as a systematic design thinking method sequence was made to redefine the value proposition in a business model. Before step 1, there might have been some systematic idea collection from the strategic documents, such as the development policy, the strategy or the organisation’s vision and mission statement, etc., and/or idea generation of new values by employees or other stakeholders.

**Fig. 5:**
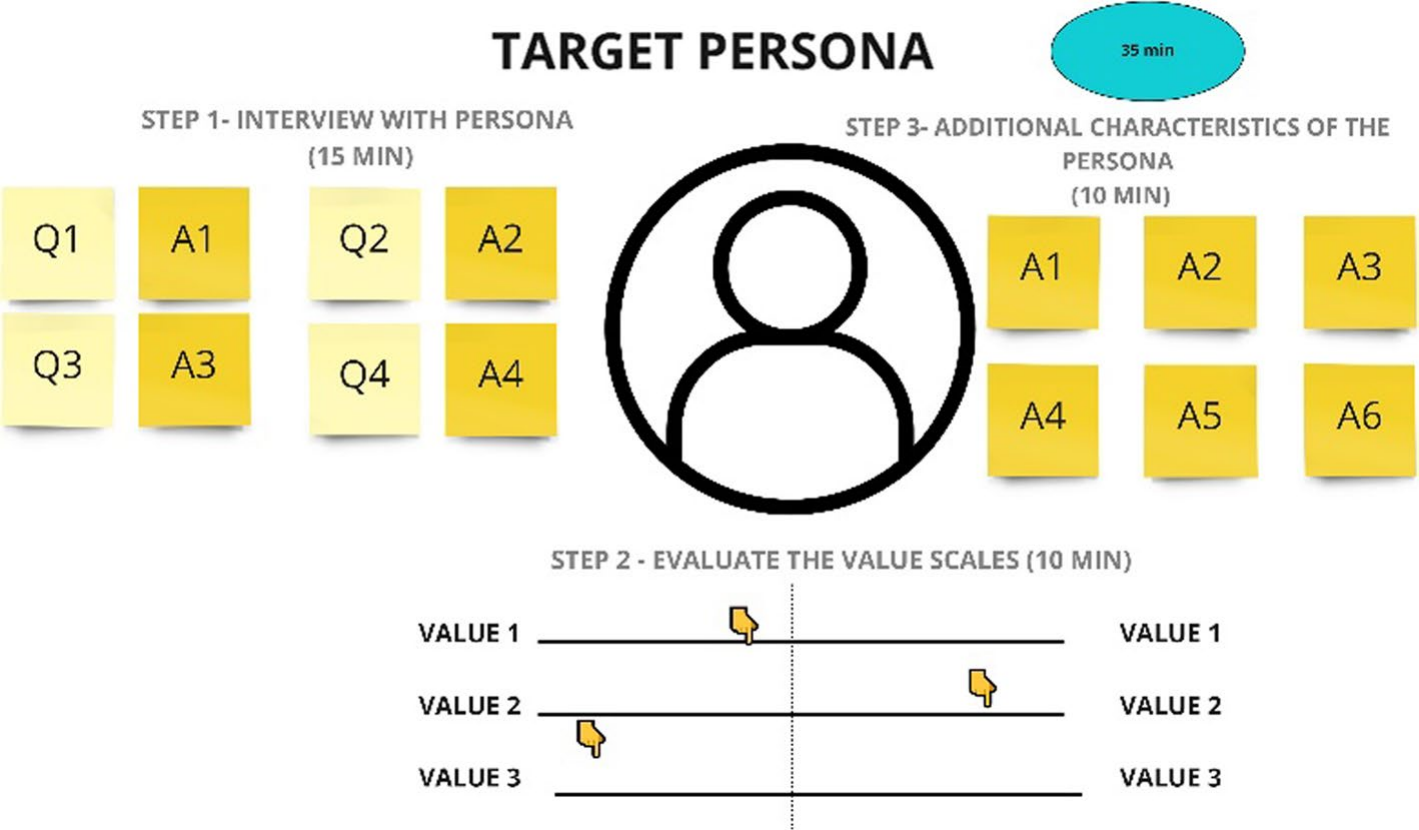
Step 1: “Warm-up” of the 4-step sequence of idea management to reinvent values (source: created by authors)

Step 1 is a warm-up (see Fig. 5), it helps to understand the customer. Based on the “Persona Canvas”, “Imagine Persona” approaches, the authors have created the first step “Target Persona”, that consists of three sub-steps. At the first step, at least 4 questions that you would like to ask to your customers about their life-styles, attitudes, etc., must be generated. After each question group, probable customers’ answers could be registered. These questions could be without a direct aim to understand the values, but just to understand the customers. For example, “What kind of vehicles do you prefer?” Based on the answers received: a bicycle, Tesla or BMW it can give the possible directions of what would be considered as a person’s values. The second step is to define contrasting values and evaluate where among them your target persona could place herself/himself. For example, this person is a playful or a serious one. The third step is aimed at identifying and recording as many additional characteristics for a target persona as you can. These could be things he/she likes, does, etc. The duration of the step is from 35 min to an hour.

**Fig. 6:**
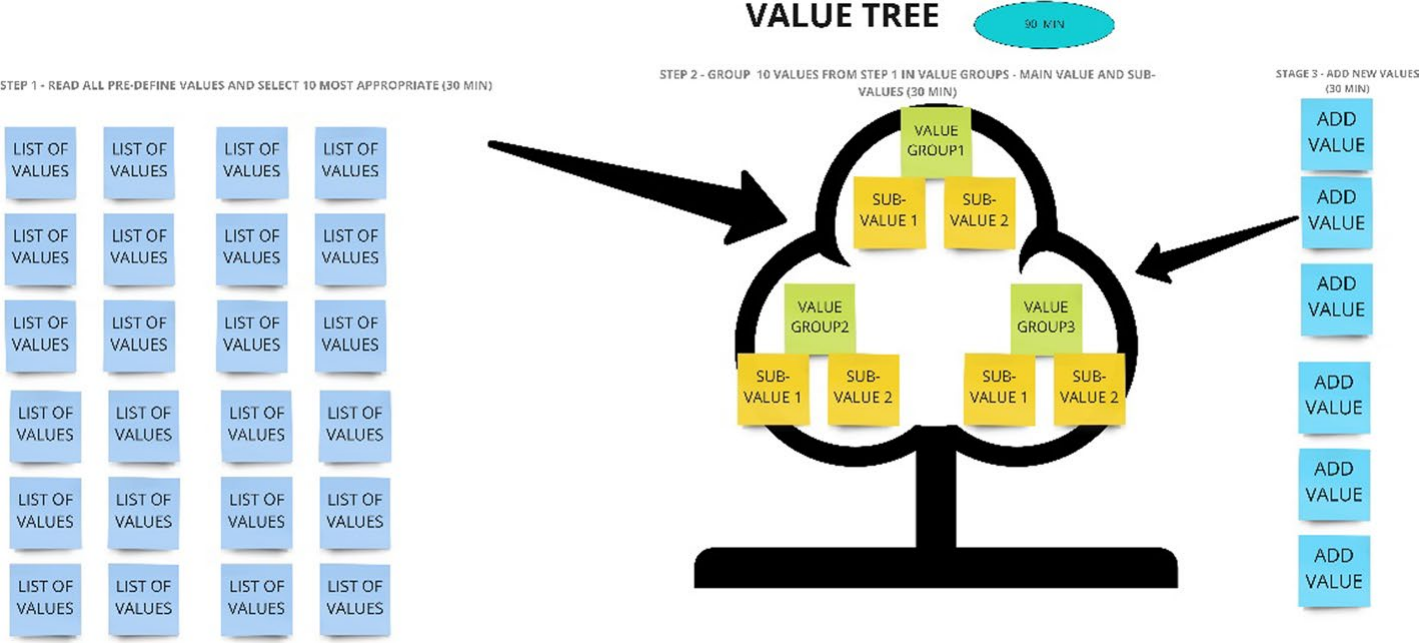
Step 2—summarise values of the 4-step sequence of idea management to reinvent values (source: created by authors)

Step 2 (Fig. 6) encompasses a collection and sorting activity where all ideas are categorised. This step identifies the ideas that apply to the task. There are two versions of this step. The first version of step 1. implies that a team has a pre-defined list of values, and they select the top 10 of the most promising values. The second version of step 1 is to generate a list of possible values (firstly, generate at least 20 values, then select the top 10 values and do not merge these processes). During step 2, the top 10 values should be grouped into three main value groups (the main value and sub-values). For example, if knowledge is the main value, then innovation and open-mindedness could be sub-values. The last step includes adding of new values and additional notes. The duration of the step is from 1 h to several hours.

**Fig. 7:**
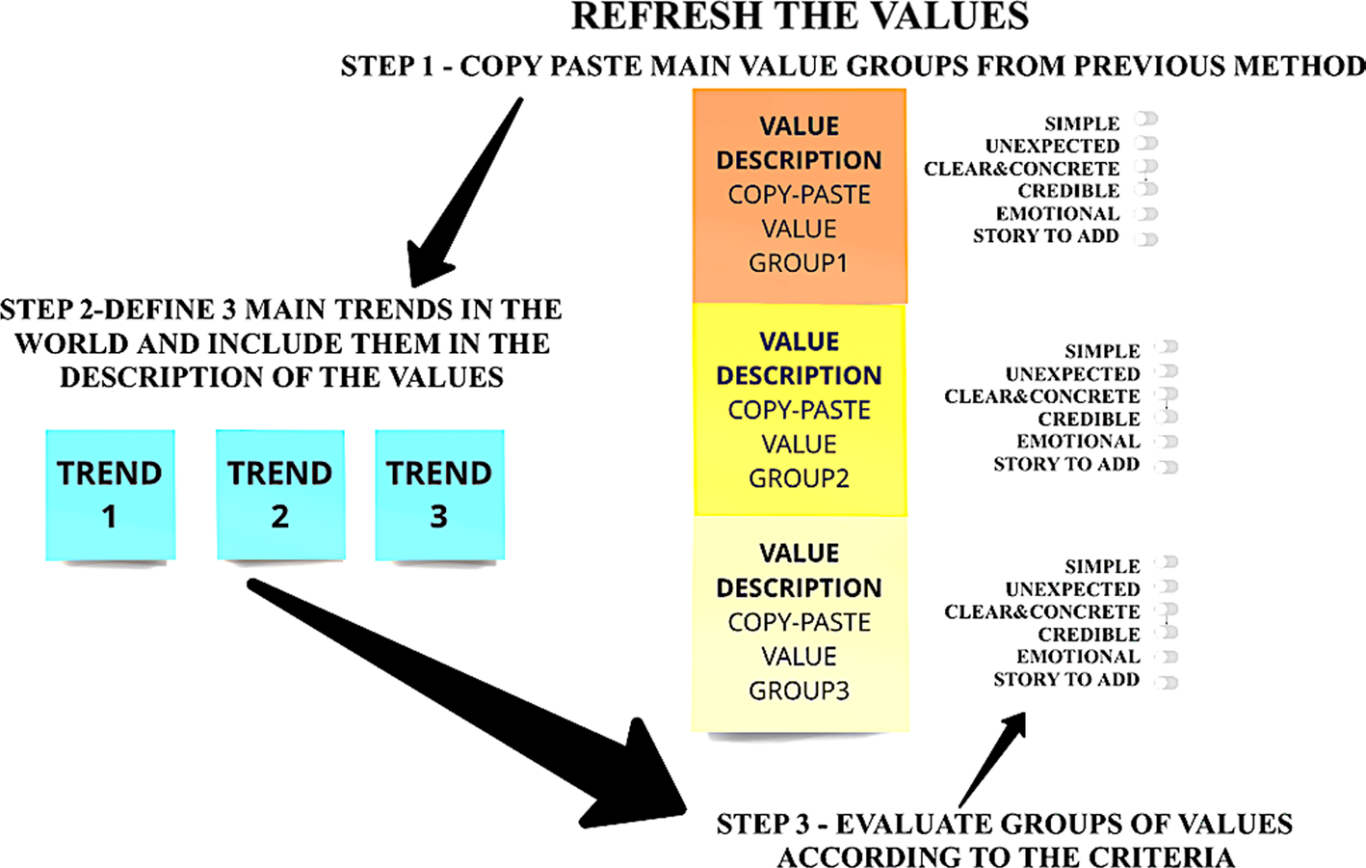
Step 3 “Enrich the value definitions” of the 4-step sequence of idea management to reinvent values (source: created by authors)

Step 3 (Fig. 7) is targeted at refreshing the definitions of values. Firstly, copy and paste the descriptions of the value groups from step 2. After that, improve the descriptions by trends (Trend watching approach), add some sentences that would show that these values are up-to-date.

Secondly, select 3 tendencies and include them in the descriptions. Thirdly, define the criteria for good value descriptions and evaluate the descriptions. In the given case 6 criteria have been adapted from Heat and Heath (2007), which encompasses six principles of sticky ideas: simplicity, unexpectedness, concreteness, credibility, emotional, stories (potential to add stories or build on these stories).

**Fig. 8:**
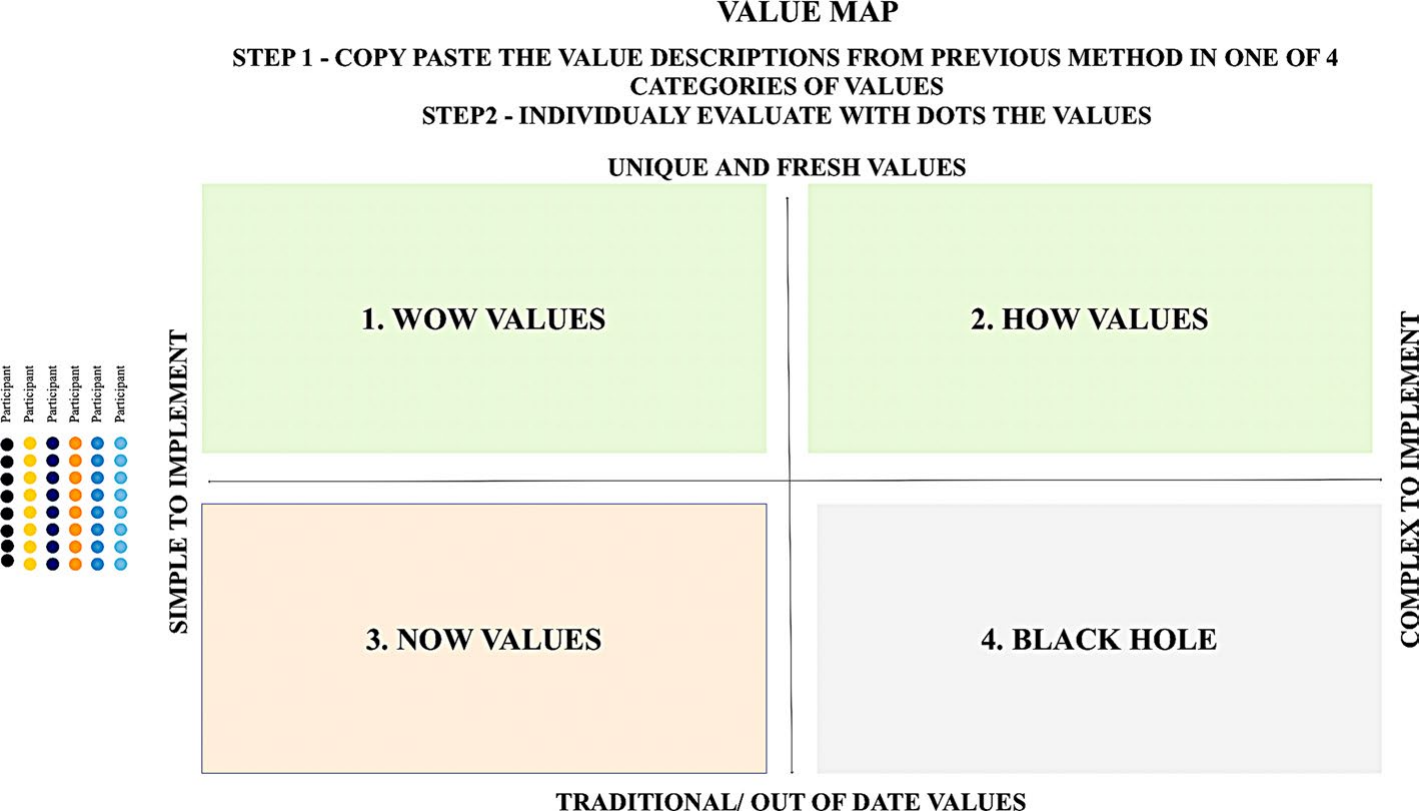
Step 4 “Evaluate the values” of the 4-step sequence of idea management to reinvent values (source: created by authors)

Step 4 (Fig. 8) is aimed at evaluating the created value descriptions. For this step, two approaches have been adapted—“How-Now and Wow” approach to evaluate ideas according to their innovativeness and simplicity. The second approach applied is “Dot Voting” approach to see the public opinion about the values. See a 4-step approach in Fig. 1. After all these steps, the post-session could be developed to give for the evaluation all created value descriptions to different stakeholders and also give criteria for evaluation.

This sequence was tested in a real company with the aim of reinventing their value proposition. During the pre-session, 74 values from strategical documents, normative acts, the visions of the company, etc., were collected; 76 employees and partners were involved in the evaluation of these values. All values were evaluated using a 5-point Likert scale.

Step 1—Warm-up: 4 groups were created and in each group there were 5 people. For this step, only 35 min were given. On average, 8 questions were defined and for each question, 4 answers were given. In the value scales, 10 pre-defined values were evaluated (all group results were consistent and not conflicting). On average, the groups recorded 12 additional characteristics of a target persona.

Step 2—Summarize the values: during this step, the teams received a list of 35 values with the highest range gained at the pre-session. They could see also the 10 values which got the most points. Though, during the selection process, the team chose values that were also not included in the top 10, so it was reasonable to give a wider perspective and the variation for the teams. During the second step, all the teams generated 3–4 groups of values and in each group 2–4 sub-values were included. Only 2–5 additional values were added by each team, so the preparation with the value collection was successful and only a few values were missed. Even the groups started with the same values—until the end of this method came up to just 3 main values that duplicated, but later the descriptions of a theme were quite different (in step 4, these values would be merged).

Step 3—Enrich the value definitions: during this step the teams copy pasted the descriptions of value groups, including the sub-values. Then these descriptions were enriched with 3 trends in the future that were important for this company. After this step, the descriptions of values were evaluated by the criteria. If the criteria were not approved, the teams tried to improve the description. Almost all value descriptions received 4 or more approved criteria.

Step 4—Evaluate the values: during this step, all created value descriptions were copied to this step and the same values were merged as one. Only 4 values were merged and then all value descriptions were evaluated according to their innovativeness and simplicity. There were no ideas in “Black hole”, so this approach led to promising value propositions. There were 2 NOW ideas—simple but traditional values, but all other values were very innovative. 6 HOW ideas that were complex for implementation, but 4 WOW ideas that were easy to implement. By using this approach, the company’s aim was reached to define 4 value descriptions that would be innovative but simple for implementation. All created descriptions were given for public evaluation and the same values got the support.

After the session, the participants and managers acknowledged it was an unexpected 8 h of a playful process that led to serious results. An additional note is that this process was moderated in Zoom and Miro environments, but it could be also moderated in a face-to-face session.

## Discussion

This paper offers a systematic 4-step sequencing process to redefine or reinvent values. The aspiration of the 4-step IM sequencing approach is to provide a more systematic view on how to get to the best possible values by creating and evaluating them. Other scholars believe that sequencing increases the engagement, interest and confidence of individuals in the group brainstorming sessions (Jimenez-Liso et al., 2022), especially it is important when the ideation sessions last for several hours or full working days as this was the case when applying the 4-step approach in practice. Meanwhile, the diversity of the content and type of tasks in a pre-planned sequence increases the creativity and the quality of the new ideas generated (Meslec et al., 2020).

The adoption of the action-based research methodological framework and results gained helped synthesise new knowledge that is available for a wider range of audience inside and outside of the organisation involved in this research. Somekh (2005) believes that this new knowledge and experience has a potential to be useful in other contexts and settings of changing situations within this organisation or even outside its boundaries.

A key practical implication is related to the possibility of using created sequences’ templates for the value creation or reinvention process. The approach may help organisation and enterprise innovators who desire to create a more systematic and playful value creation process. As a result, a decision-maker will have more values to choose from while inventing new or reinventing existing values in their business models. The IM may provide far more quality and playfulness to the complex, innovative processes of inventing new and reinventing established values in business models. Other scholars confirm that; besides the quality and game dynamics, the IM may enhance the efficiency of the ideation process (Hesmer et al., 2011).

A key theoretical implication is related to the new combination and modification of the IM approaches to adapt them to this specific aspect of the business model and a value reinvention. It may be possible to include this 4-step approach as one perspective for understanding how to design and develop new values for the business model.

Further research could explore the potential and effectiveness of diverse stakeholder involvement in the value creation or reinvention process. The additional insight from more diverse stakeholder involvement may lead to additional insights that can play an important role in the value creation or a redefinition process.

A philosophical implication is related to the dominating assumption about the role of the IM in a business model development and innovation processes, since mostly it is related to the first stages of the innovations (Gerlach & Brem, 2017; Herrmann et al., 2020; Sandriev & Pratchenko, 2014; Sandstrom & Bjork, 2010). We may need to reconsider the IM as a part not only to create business models but also as the process that could keep it up-to-date all the time or maintaining the status quo and create sustainable competitive advantage (Vandenbosch et al., 2006).

An additional question is how this approach works in real-life sessions, because in this case, it was applied in a web-based session. The results were very good, but there are a lot of discussions that technologies destroy creativity (Edwards, 2001; Hoffmann et al., 2016; Todd, 2003), but maybe in a systematic and well-managed IM process, creativity could be boosted, as it may facilitate more open discussion and co-working environment.

In the current contribution, one sequence of the 4-step method is applied. In other sequencing research with other methods, the importance of the length of the sequence is highlighted (Meslec et al., 2020). Future research should focus on the effectiveness of longer and shorter sequences of IM methods to reinvent the values for business models. In future studies, authors might create alternative concepts for sequencing and test other sequences of IM methods. Additional research should be conducted to explore the effectiveness of the created sequence. Also, comparative research could be made to compare this approach to others.

Authors developed and tested this approach in a single case. The test with sequencing could be done in different industries—to reveal if this approach holistically applies or fits only for specific industries and cases.

## Conclusions

### Theoretical implications

This paper offers several theoretical implications for scholars and researchers. First, the business models and value creation have been used in a variety of research contexts, but it has not been extensively applied to the field of IM. This study provides a useful framework for the IM application within the business model value creation context and detailed characterisation of practical construct. The study creates a theoretical and practical framework. It expands the domain of the IM by characterising importance not only of sequencing but also the diversity and interrelation of methods.

Second, results provide some insights that may help in designing future studies. They highlight the importance of empirical and theoretical research to select elements to include in such frameworks. It also shows that there are a lot of possible elements to research in the future. In future studies, researchers should evaluate and select the most appropriate methods for focused studies.

Main theoretical implication is conceptualisation and testing of the 4-step IM sequencing approach to redesign or reinvent the values for business models. In addition, using an action-based research methodological approach allows to identify main practical conclusions, and, also, immediately receive feedback and verify them with individuals involved.

Regarding the IM concept perspective, this study contributes to the theoretical and practical proof of the importance of the IM sequencing with diverse methods for different IM stages.

Regarding the business models’ literature perspective, this article shows how sequencing of the IM methods could contribute to the creation and re-invention of business models and the values.

The application of the IM methods in the existing remote work environment has created new challenges for the virtual moderation of design thinking and ideation sessions. Digital tools help to develop attractive and engaging methods for active participation within the ideation sessions of new values. The design sprint and gaming elements integrated within the 4-step approach help to keep the attention and engagement of participants in longer (e.g. 8-h) ideation sessions.

Our findings reveal that this 4-step sequencing approach of the IM methods enhances the participants’ ability to reinvent values. Also, it proved the persistent interest, engagement, confidence, and trust of involved participants.

### Practical implications

Main practical contribution is the highlight of practically applicable IM sequence for reinvention of values. Researchers have published all practical method templates that could be used in various organisations to apply the method sequence in their work. Created sequence could be used both in real-life sessions and on web-based tools.

The action research approach applied allowed the close cooperation between researchers and practitioners to maximise the results of the ideation sessions. It stimulates the development of new knowledge among the practitioners on the IM applied. The participants of the 4-step approach for reinventing the values became as the knowledge ambassadors on these methods and may further promote these approaches within their organisation.

The 4-step approach to systematic and structured tasks encourages more effective ideation, leading to new ideas of values. The variation of the length and type of tasks bring entertaining aspects, keeping participants interested in the process and contributing to the ideation.

### Scope of future research

There have been three main limitations: (1) analysed literature sources amount based on the research design (selected databases, time frame and selection approach); (2) application of only 4 IM methods; (3) only one organisation involved in this action research and a case study. Based on the limitations, authors have developed suggestions for future studies:

- For future studies, scholars may create alternative concepts for sequencing tasks and test other sequences of the IM methods.
- In future studies, researchers may experiment with a diverse length of sequencing tasks to research the most appropriate length of each sequent task of the IM in order to see if 4 steps are enough. Here, authors adapted 4-step approach to the specific case—given by the company.
- Additional research should be conducted to explore the effectiveness of the created sequence. Also, the comparative research could be made to compare this approach against other approaches.
- The test with sequencing tasks could be done in different industries—to reveal if this approach holistically applies or is more appropriate for specific industries and cases.

In future study authors plan to attract experts to validate the created sequence, involving experts that represent not only the IM and business model disciplines, but also practitioners and representatives of companies that are responsible about reinvention of the values. This will allow to balance the theoretical findings with the practitioner's views. The authors believe that this paper will stimulate scientific discussions of the academic community and further research about the IM sequencing.

## Data Availability

All research data are openly accessible within the Idea Innovation Institute. To access data contact: mikelsone.elina@gmail.com.
